# Protective effects of physical activity on episodic memory during aging are explained by executive functioning

**DOI:** 10.1186/s11556-024-00341-y

**Published:** 2024-03-09

**Authors:** Ilona Moutoussamy, Laurence Taconnat, Lucie Angel, Kristell Pothier, Lucette Toussaint, Séverine Fay

**Affiliations:** 1 Département de Psychologie, Centre de Recherches Sur La Cognition Et L’Apprentissage (UMR-CNRS, Université de Tours Et de Poitiers, Centre National de La Recherche Scientifique, 37000 Tours, France 3 rue des Tanneurs,; 2 Département de Psychologie, Psychologie Des Âges de La Vie Et Adaptation (EA 2114), Université de Tours, Tours, France; 3 Département Des Sciences du Sport, Centre de Recherches Sur La Cognition Et L’Apprentissage (UMR-CNRS 7295), Université de Tours Et de Poitiers, Centre National de La Recherche Scientifique, Poitiers, France

**Keywords:** Cognitive reserve, Executive functions, Physical activity, Episodic memory

## Abstract

Aging is marked by a memory decline related to an executive function decline. Physical activity (PA) has beneficial effects on both executive functions and memory, especially in aging. The protective effects of PA on these two cognitive abilities have always been studied separately, despite the well-established relationship between memory and executive functions. Our objective was to explore whether the benefits of PA on memory could be explained by reduced age-related changes in executive functions.

Nineteen young adults (27.16 years old) and 25 older adults (69.64 years old) performed a resource-dependent memory task, three executive tasks and completed a PA questionnaire (measuring sports and leisure PA). Age group and PA effects on memory and executive performance were analyzed with generalized linear models. Mediation analyses were calculated using method of causal steps approach with a non-parametric bootstrapping procedure.

The results confirmed the effects of age and PA on memory and executive performance. A significant interaction confirmed the protective effect of PA on age-related cognitive performance. PA was positively correlated with performance in both memory and executive tasks, but only in the older adults. Although each predictor alone (age, executive functions and PA) significantly explained memory performance in older adults, only the effect of PA on memory performance remained significant when all the predictors were introduced in the analyses.

PA mediates the effects of age and executive functions on memory performance. This suggests that PA protects older adults against memory decline by reducing the decline in executive functioning.

## Introduction

Understanding the accompanying changes and the protective factors against cognitive aging has become a major health issue. Impaired episodic memory (involving retrieval of personal experiences and their spatial and temporal contexts) is one of the main concerns of older adults, and is well documented in the literature (see [1] for a meta-analysis). Episodic memory loss is greater in tasks that do not provide environmental support (e.g., free recall, [2–4]) and require participants to self-initiate efficient memory strategies. These tasks require more executive resources than those that provide environmental support (e.g., recognition).

Executive functions are defined as “a set of general-purpose control mechanisms, often linked to the prefrontal cortex of the brain, that regulate the dynamics of human cognition and action” [5]. These specific functions are involved in self-initiated memory strategies during encoding and retrieval (e.g., [6]). Several studies and meta-analyses have confirmed that executive functioning is impaired in aging, with a deficit in each specific function presented in Miyake et al.’s [7] model (i.e., inhibition, flexibility and updating; see [8] for a review). According to the executive hypothesis of aging [9], the age-related deficit in executive functions could explain episodic memory decline, especially in the most resource-demanding tasks, due to difficulty in self-initiating effective memory strategies [6, 10, 11].

Many individual differences in episodic memory performance can be observed among older adults, both qualitatively (e.g., vivacity and precision of recall) and quantitatively (e.g., number of correct recalls). These differences can be explained by cognitive reserve, defined as mechanisms that protect against cognitive decline [12]. Cognitive reserve allows older adults to compensate for decline through a more efficient use of cognitive processes. Thus, healthy older adults with high cognitive reserve show less episodic memory decline. Factors such as educational level, social and physical activities have been studied for their protective effects on episodic memory in advancing age [13].

Voelcker-Rehage et al. [14] showed that physical activity (PA) (grip force and spiroergometry, i.e., cardiovascular fitness) was positively related to performance on a modified version of the Flanker Task and N-Back task in older adults (see [15] for a meta-analysis). PA (measured with actimetry) has also been found to be positively associated with episodic memory performance, but only in older adults (e.g., [16], see [17, 18] for two meta-analyses). These results have been replicated with self-report measures, such as PA questionnaires (see [19, 20]). In most studies, PA is seen as a protective factor during aging, as its benefits are observed more in older than in younger adults. Moreover, these benefits seem to depend on the complexity of the task, as they appear to be greatest on the most demanding episodic memory tasks [21, 22].

Various explanatory factors of the benefits of PA on cognition have been put forward, including neurobiological factors (e.g., functional and structural brain changes or cellular and molecular changes; see [23, 24] for reviews) and cardiovascular factors (e.g., [25]). However, the benefits of PA on cognition are not consistent across studies, and multiple factors may be involved in these discrepancies. The neurobiological and cardiovascular factors alone cannot explain for these differences which may be due to complementary cognitive mechanisms. Some researchers have suggested that these benefits could be explained by a lower decline of executive functioning (e.g., [20], but see [26] for a model of acute PA on memory function). According to this view, physically active older adults would experience a lower decline of executive functions, thereby maintaining effective memory. However, no study has directly tested this hypothesis.

Many studies have shown the protective effect of PA on episodic memory and executive functions, as well as the association between episodic memory and executive functions. No studies have examined the relationships between all these variables. While some authors have suggested that executive functions might mediate the effect of chronic PA on episodic memory, this has never been tested (see [26] for a model suggesting the same executive hypothesis of acute PA on memory function). Thus, the aim of the current study was to determine whether the beneficial effects of PA on a resource-dependent episodic memory task could be explained by a lower decline of executive functioning in the aging population. We aimed to confirm that (1) memory and executive performance would be better in younger than older adults, and (2) PA would be beneficial for both memory and executive performance, particularly in older adults. Finally, if these prerequisites are met, we expected that PA would be the strongest predictor of memory performance in older adults, and that this factor would mediate the age and executive effects on memory performance. Based on these empirical results, a model of the benefits of PA on memory explained by executive function in older adults could be proposed.

## Material and methods

### Participants

Nineteen young adults (aged 21 to 39 years) and 25 older adults (aged 61 to 88 years) participated voluntarily in this study. All the participants were French-speaking, had no anxiety or depressive disorders (score < 11 on each subscale of the HADS [27]), no neurological or psychiatric history, and were not taking medication that could affect cognition. The older adults were screened on the Mini Mental State Examination (MMSE [28]); they all scored over the 27-point cut-off. Participants’ characteristics are presented in Table 1. Younger adults had a higher educational level than older adults [*t(42)* = 4.49; *p* < 0.001] (measured by the number of years of education associated with the highest degree obtained). No significant differences between our groups were found in terms of vocabulary [*t(42)* = 1.17; *p* = 0.249] (measured with the Mill-Hill [29]), anxiety [*t(42)* = -1.04; *p* = 0.305], depression [*t(42)* = -1.14; *p* = 0.259] (both measured with the HADS) or gender [Pearson Chi^2^ = 1.83; *p* = 0.176]. This study was approved by the ethics committee of the University of Tours (France).

**Table 1:** Means (and SD) of participants’ characteristics in each age group

	**Younger adults (*n* = 19)**	**Older adults (*n* = 25)**
**Age** (years)	27.16 (5.29)	69.64 (9.37)
**Educational level** (years)	13.32 (2.14)	10.44 (2.08)
**Vocabulary** (maximum score 34)	23.84 (2.48)	22.28 (5.40)
**Anxiety** (maximum score 21)	3.63 (2.19)	4.44 (2.80)
**Depression** (maximum score 21)	3.95 (2.59)	4.92 (2.94)
**Sex ratio** (% of women)	31.58	52.00

### Material and procedure

The procedure was carried out in the following order: consent form, MMSE for older adults, Stroop task, familiarization (3 trials) and episodic memory task, Trail Making Test, 2-Back test, and finally HADS, Mill-Hill and PA questionnaire.

### Episodic memory task

**Fig. 1:**
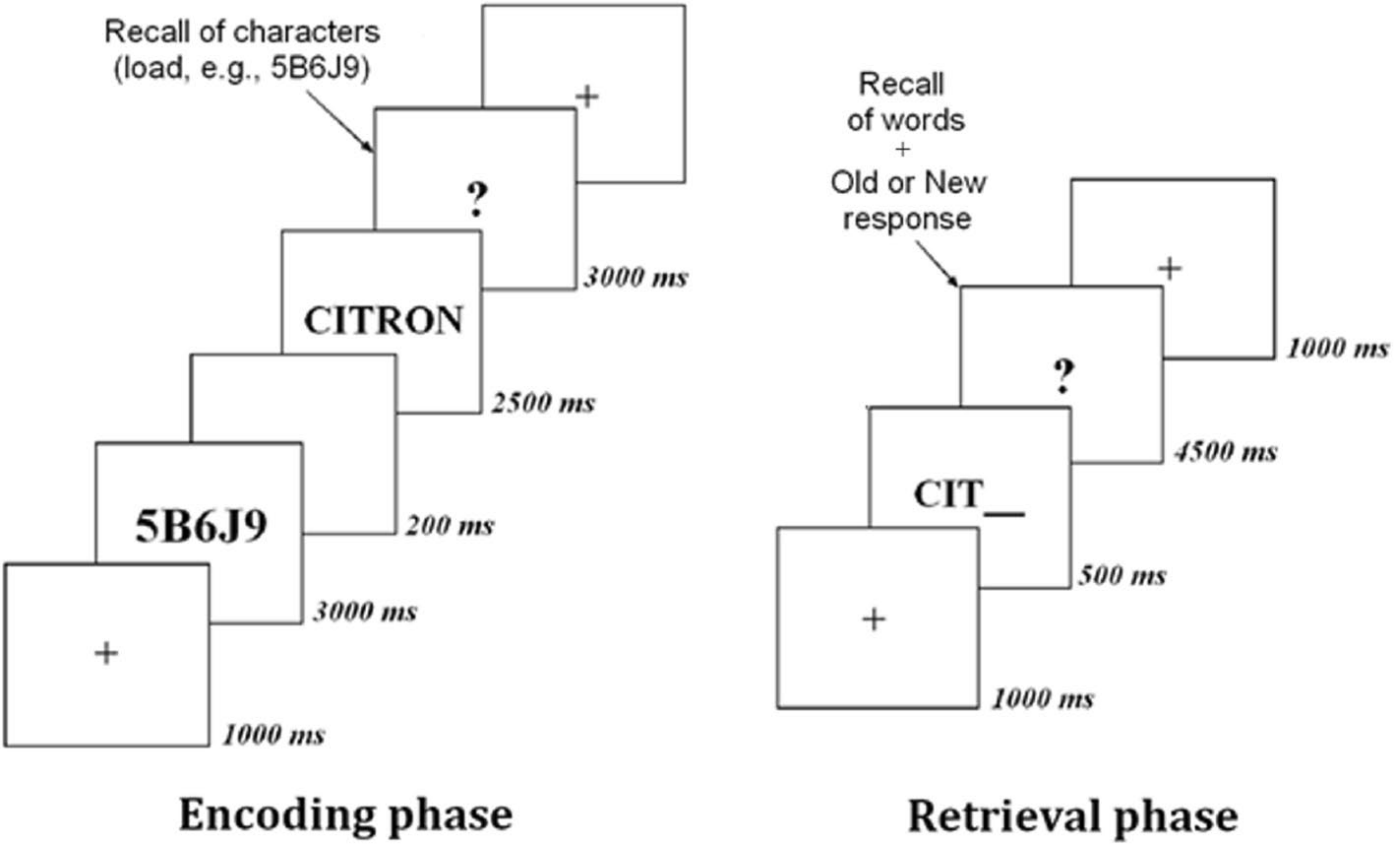
Procedure of the episodic memory task for both encoding and retrieval phases

Following Moutoussamy et al. [20], we used a resource-dependent paradigm, in which a high-load working-memory task was introduced during the encoding phase of a word-stem cued recall task (episodic memory task, Fig. 1). Before the encoding of each target word, participants had to memorize in working memory a series of 5 random characters, alternating letters (from B to L, without vowels) and numbers (from 1 to 9), always starting with a number. No letter or number appeared twice to avoid chunking. This load must be recalled after the presentation of the word to ensure that it has been maintained in working memory during the word encoding. The episodic memory task involved three lists of ten words (counterbalanced), two for the encoding phase, and an additional one for the retrieval phase. Thus, the participant completed 30 trigrams during the retrieval phase. The words in each list were similar in terms of length (*M*_1_ = 8.10 ± 1.12; *M*_2_ = 8.10 ± 1.52; *M*_3_ = 8.00 ± 1.03; *F*(2,57) = 0.04, *p* = 0.96), frequency of use in French corresponding to the average occurrence in books and films (*M*_1_ = 2.07 ± 2.19; *M*_2_ = 2.47 ± 2.80; *M*_3_ = 2.83 ± 3.56; *F*(2,57) = 0.35, *p* = 0.71), and concreteness (evaluated by 57 volunteers aged 20 to 64 on a five-point scale, with one corresponding to very abstract nouns and five to very concrete nouns, *M*_1_ = 3.69 ± 0.99; *M*_2_ = 3.77 ± 0.89; *M*_3_ = 3.70 ± 0.76; *F*(2,57) = 0.05, *p* = 0.95).

The procedure was as follows. In the encoding phase, a fixation cross was presented for 1000 ms, followed by a series of letters and numbers (load, e.g., 5B6J9) for 3000 ms, a blank screen for 200 ms, the word to memorize for 2500 ms, and a question mark for 3000 ms. When the question mark appeared, participants had to recall the series of letters and numbers. To ensure that none of the participants had neglected the working-memory task (i.e., recall of the series of letters and numbers), only those with more than 70% correct responses were retained for statistical analysis. In the retrieval phase, a fixation cross appeared for 1000 ms followed by a word-stem for 500 ms and a question mark for 4500 ms. When the question mark appeared, participants had to recall a previously learnt word starting with the stem, or produce a new word beginning with the same letters and they had to say whether the produced word was an old or a new one. Theoretically, the whole task required a high level of executive control, as the working-memory task plus the episodic-memory task place a high demand on cognitive resources [30, 31]. A three-by-three countdown task is performed between encoding and retrieval, for a duration of 15 s to avoid any recency effect.

Two dependent variables were used: the percentage of incorrectly recalled letters/numbers (i.e., working-memory task) and the percentage of correctly recalled words (i.e., episodic memory performance). The episodic memory performance represents a conscious retrieval of the memory in its learning context since it represents the percentage of trigrams of old words completed by an old word and judged as old by the participant. For example, “*CITRON*-Old” is a conscious retrieval but “*CITRON*-New” is an unconscious retrieval and did not account in the percentage of correctly recalled words.

### Executive functions: updating, inhibition and flexibility

Updating was assessed with a 2-Back task [32]. Thirty letters were presented aloud by the experimenter at a frequency of one letter per second. For each letter, the participant had to indicate whether it was the same as the one given two letters before, answering "yes" or "no". A practice session was carried out first, to ensure that the instructions were understood. The number of correct answers was counted (maximum 28).

Inhibition was evaluated using a paper-and-pencil Stroop task [33]. Three cards were presented for 45 s each. On card A, color names were written in black ink and the participant had to read a maximum number of words. Card B showed crosses printed in red, green or blue ink, and the participant had to name a maximum number of colors. On card C, color names were written in a different ink color, and the participant had to name as many ink colors as possible, inhibiting reading the word (e.g., when the word blue is printed in red, the correct answer is red). The number of items correctly completed in 45 s for each card was counted. The inhibition index was calculated by subtracting the result of card C from that of card B and dividing the result by the score of card B (i.e., [B minus C] divided by B) [34]. This gives a value between 0 and 1: the lower the index, the better the performance.

Flexibility was measured with a paper-and-pencil Trail Making Test [35]. Participants are shown two cards. Card A has numbers from 1 to 25, and the participant has to draw a line connecting them in ascending order as quickly as possible. Card B has numbers (from 1 to 13) and letters (from A to L), and participants have to link the numbers and letters alternately in ascending order (1, A, 2, B, etc.) as quickly as possible. The flexibility index was calculated by subtracting the time taken to complete card A from the time to complete card B (i.e., TMT B minus TMT A) [36]. The lower the index, the better the performance.

Finally, a composite index comprising the three measures was calculated for each participant to obtain an index of overall executive functioning. The load factor of each executive function was calculated using factor analysis (-0.75 for inhibition, -0.90 for flexibility, 0.91 for updating), confirming their involvement in a common factor, the executive index (0.74 for total load factor). Each executive function performance was transformed into a *Z*-score before the following ponderation formula was applied. A higher index indicates a higher level of executive functioning.

$$executive \,index =\sum_{i=1}^{3}[Z \,score \,of \,executive \,performance \,x \,load \,factor \,for \,each \,measure]/total \,load \,factor$$

### Chronic PA questionnaire

Baecke’s questionnaire [37] was used to assess physical and sports activities (e.g., yoga, swimming, dance) and daily activities (e.g., walking, moving about). This questionnaire measures three domains of daily PA over the last 12 months: PA at work (IAT, 8 questions), PA during leisure time (IAL, 3 questions) and sports (IAS, 2 questions). The physical activity and sport index (IAS) is calculated using the frequency, intensity and duration of the two most practiced physical activities, to account for differences in intensity between individuals. Like Bigard et al. [38] and Moutoussamy et al. [20], we only assessed IAL and IAS, since the majority of older adults are no longer working. The questionnaire and the calculation formula are described in detail in Moutoussamy et al. [20]. The overall PA index is the sum of IAL and IAS: the higher the index, the greater the level of daily PA.

### Statistical analyses

All the statistical analyses were conducted using R 4.2.1 software. Age group and PA effects were analyzed with generalized linear models (GLM) for each dependent variable (i.e., % correctly recalled words, % errors on the working-memory task, and executive index). Effect sizes for significant effects and interactions were calculated using partial eta squared (η_p_^2^). As PA was analyzed as a continuous variable in the GLMs, correlation analyses were conducted between PA and cognitive measures to understand the significant effects and interactions. Finally, based on the relationships observed between our variables in each age group, mediation analyses were calculated using method of causal steps approach (i.e., hierarchical linear regression analysis). A non-parametric bootstrapping procedure was used as some predictors do not follow a normal distribution in older adults. Coefficients (b) and confidence intervals (CI) were estimated with 5000 bootstrap resamples. As recommended by Wood et al. [39], we tested the significance of indirect effects using Preacher and Hayes’ [40] bootstrapping process for R version 4.1.1. Thus, when zero is included in the confidence interval, the effect cannot be considered as significant. By contrast, when zero is not included, the effect can be considered as significant.

## Results

Results for the episodic memory task, executive functions and PA are presented in Table 2. No effect of age group on IAL and IAS [*t(42)* = 1.47; *p* = 0.149 and *t(42)* = -0.28; *p* = 0.781 respectively] or on the overall PA index [*t(42)* = 0.03; *p* = 0.975].

**Table 2:** Means (and SD) of memory scores, executive index and PA measures for each age group

	**Younger adults (*n* = 19)**	**Older adults (*n* = 25)**
**Episodic memory score**		
% of correctly recalled words	48.16 (10.57)	25.00 (10.41)
**Working-memory score**		
% of errors on the working-memory load	10.53 (6.64)	21.00 (6.12)
**Executive functions scores**		
Inhibition	0.32 (0.11)	0.448 (0.09)
Flexibility	26.05 (7.10)	59.04 (32.96)
Updating	24.68 (1.83)	21.40 (3.37)
Executive index	2.23 (1.37)	-1.53 (2.68)
**Physical activities**		
Leisure activities (IAL)	2.74 (0.65)	3.08 (0.84)
Sports (IAS)	4.27 (3.54)	3.97 (3.64)
PA index	7.01 (3.52)	7.05 (3.91)

### Episodic memory

The GLM conducted on episodic memory performance confirmed the age-group effect, *F* (1,40) = 90.85, *p* < 0.001, η_p_^2^ = 0.69, with fewer words correctly recalled by older than younger adults. The results also showed an effect of PA, *F* (1,40) = 17.07, *p* < 0.001, η_p_^2^ = 0.30. Correlational analyses indicated that PA was positively related to episodic memory performance (*r* = 0.32, *p* = 0.034). Finally, the interaction between age group and PA was significant, *F* (1,40) = 15.28, *p* < 0.001, η_p_^2^ = 0.28. More precisely, to investigate this interaction, correlation analyses were conducted and indicated that the positive correlation between PA and memory performance was significant in older adults (*r* = 0.89, *p* < 0.001) but not in younger adults (*r* = -0.10, *p* = 0.695).

The GLM performed on the working-memory task revealed an effect of age group, *F* (1,40) = 35.43, *p* < 0.001, η_p_^2^ = 0.47, with a higher percentage of errors by older than younger adults. This analysis also showed an effect of PA, *F* (1,40) = 10.14, *p* = 0.003, η_p_^2^ = 0.20; participants with higher levels of PA made fewer errors on the working-memory task (*r* = -0.34, *p* = 0.024). This effect was similar in younger and older adults (respectively, *r* = -0.28, *p* = 0.244 and *r* = -0.57, *p* = 0.003), as there was no significant interaction between age and PA, *F* (1, 40) = 0.56, *p* = 0.458.

### Executive index

The GLM performed on the executive index revealed an age-group effect, *F* (1,40) = 44.52, *p* < 0.001, η_p_^2^ = 0.53, with better executive functioning in younger adults. The analysis also showed an effect of PA, *F* (1,40) = 15.88, *p* < 0.001, η_p_^2^ = 0.28, with higher levels of PA corresponding to a higher executive index (*r* = 0.39, *p* = 0.010). Finally, a significant interaction was found between PA and executive index, *F* (1, 40) = 4.57, *p* = 0.039, η_p_^2^ = 0.10, indicating that this relationship was significant only in older adults (*r* = 0.63, *p* = 0.001) and not in younger adults (*r* = 0.24, *p* = 0.315).

### Regression analysis

**Fig. 2:**
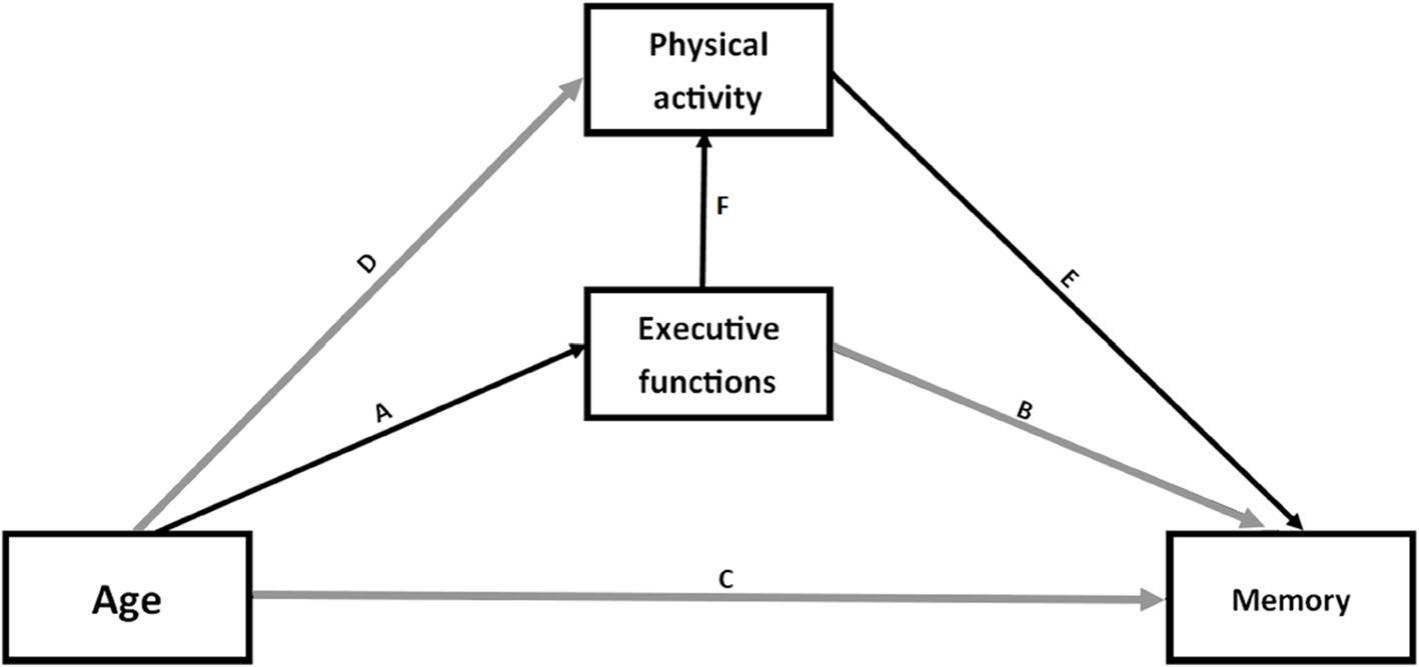
Mediation model and paths in older adults explaining episodic memory performance with age, PA and executive index as predictors. An arrow indicates that the effect of the first variable is mediated by the second variable (indicated by the arrowhead). Main findings: ABC indicates that the effect of age on memory could be predicted by executive functions. DEC indicates that the effect of age on memory could be predicted by PA. The significant indirect pathway (in black) indicates the effect of age on memory could be predicted by executive functions, which itself could be predicted by PA

Episodic memory performance and executive index were positively correlated (*r* = 0.60, *p* = 0.002) in older adults. Significant negative correlations were found between these two variables and the chronological age of older adults (*r* = -0.50, *p* = 0.01 for episodic memory, *r* = -0.67, *p* < 0.001 for executive index) and between PA and the chronological age of older adults (*r* = -0.56, *p* = 0.004 for PA). As these linear relationships were only found in older adults, regression analyses were performed only in this age group to better understand the underlying mechanisms of PA benefits on memory and executive functions in old age. The mediation model (including age, executive index and PA as predictors of memory performance) and the different pathways are presented in Fig. 2.

First, the regression analysis revealed a significant direct association between age and episodic memory performance (i.e., percentage of correctly recalled words), *F* (1,23) = 7.84, *p* = 0.010, explaining 25.4% of the variance. Age was negatively associated with memory performance (path C, *b* = -0.56; 95% CI [-0.88, -0.19]).

The simple mediation model predicting memory performance from age and executive index (paths A, B and C) was significant, *F* (2,22) = 6.70, *p* = 0.005, explaining 38% of the variance in memory performance. To go further, age was negatively associated with executive index (path A, *b* = -0.19; 95% *CI* [-0.29, -0.010]), and executive index was positively associated with memory performance (path B, *b* = 2.33; 95% *CI* [0.81, 3.34]). After controlling for executive functioning (*b* = 1.85; 95% *CI* [0.15, 3.12]), the negative association between age and memory performance was no longer significant (*b* = -0.20; 95% *CI* [-0.64, 0.20]). Importantly, the indirect effect explaining the effect of age on memory performance through executive index was significant, 95% *CI* [-0.76, -0.11].

The other simple mediation model predicting memory performance from age and PA (paths D, E and C) was also significant, *F* (2,22) = 40.37, *p* < 0.001, explaining 78% of the variance in memory performance. To go further, age was negatively associated with PA (path D, *b* = -0.23; 95% *CI* [-0.34, -0.11]) and PA was positively associated with memory performance (path E, *b* = 2.36; 95% CI [1.81, 2.76]). As in the previous model, after controlling for PA (*b* = 2.33; 95% *CI* [1.77, 2.85]), the negative association between age and memory performance was no longer significant (*b* = -0.02; 95% *CI* [-0.26, 0.24]). For this model, the indirect effect explaining the age effect on memory performance through PA was significant, 95% *CI* [-0.81, -0.31].

The overall mediation model predicting memory performance from age, executive index and PA was significant, *F* (3,21) = 26.23, *p* < 0.001, explaining 79% of the variance in memory performance. To go further, the executive index was positively associated with PA (path F, *b* = 0.91; 95% *CI* [0.52, 1.32]). Most importantly, the effects of age and executive index on memory performance were no longer significant (respectively, *b* = 0.03; 95% CI [-0.28, 0.34] and *b* = 0.34; 95% CI [-0.92, 1.35]) when PA (*b* = 2.25; 95% CI [1.68, 2.80]) was entered in the regression analysis. These important results show that the effects of both age and executive index on episodic memory are mediated by PA. In this mediation model, the indirect effects explaining the effect of age on memory performance through executive index (paths A and B) and through PA (paths D and E) were no longer significant, 95% *CI* [-0.28, 0.17] and 95% *CI* [-0.46, 0.07] respectively. The serial indirect pathway explaining the effect of age on memory performance through executive index and then PA (corresponding to paths A, F and E) was significant, 95% *CI* [-0.68, -0.06], suggesting that the effect of age on memory could be predicted by executive index, which itself could be predicted by PA.

As PA was also positively associated with executive index (reverse path F, *b* = 0.43; 95% CI [0.23, 0.64]), we tested the serial indirect pathway explaining the effect of age on memory performance through PA and then executive index (corresponding to paths D, reverse F and B). This indirect pathway was non-significant, 95% *CI* [-0.10, 0.05].

## Discussion

Our main objective was to demonstrate that PA mediates both the effects of age and executive functions on the memory performance of older adults. Our results suggested that the benefits of PA on episodic memory can be explained by a lower executive decline. This is the first study to explore the effect of PA on an innovative episodic memory task that includes a high-load working memory task during the encoding phase, to highlight the importance of benefits on the strategic phases of memory recall. During encoding, the amount of executive resources available to encode the target words are reduced by a working-memory load. Theoretically, only participants with high executive resources could correctly encode the words presented. In addition, during the retrieval, participants specified an old/new judgment in order to identify episodic responses, for which the effects of PA seemed to emerge significantly.

Our results confirmed the effects of age on memory [1] and on the working-memory load [41]: younger adults correctly recalled more words and made fewer errors in the working-memory task than older adults. Interestingly, the results confirmed that PA could be beneficial for memory performance (e.g., [16, 19–22]), and showed that these benefits are not affected by a simultaneous resource-demanding task (i.e., working-memory task), which has not been demonstrated in previous studies. Moreover, the benefits of PA on episodic memory performance were only significant in older adults. Thus, engagement in PA maintains the episodic memory performance of older adults, in accordance with previous research (e.g., [16, 17]). The current study also confirmed the well-established effect of age (see [8] for a review) and the benefits of PA on executive functions, particularly for older adults [15]. Indeed, the age effects were lower in the most physically active older adults. In other words, physically active older adults had better executive performance than their less physically active peers. That confirms the protective effect of PA on episodic memory and executive functions in older adults and that PA is a cognitive reserve factor for older adults [15, 17–20].

Our main objective was to determine whether the benefits of PA on episodic memory in older adults could be explained by executive functioning in a cross-sectional study design. We expected that PA would be the strongest predictor of memory performance and that this factor would mediate the effect of age and executive functions on memory performance. The results did reveal that each predictor (age, executive functions and PA) explained the memory performance of older adults. Moreover, executive functions mediated age-related effects in memory performance. Thus, memory performance during aging could be explained by executive performance, as older adults with a higher executive index had the best memory performance, and conversely. This is in line with the results of other studies [6, 10, 11, 42–44] and is consistent with the executive hypothesis of aging [9]. More importantly, our results revealed that PA was the main predictor of memory performance and that it mediated the effects of both age and executive functioning on memory performance.

While many studies have demonstrated the benefits of PA on cerebral reserve, explaining cerebral plasticity through structural, functional or neurochemical factors (e.g., [24, 25, 45]), only a few studies have investigated the complementary cognitive mechanisms underlying the benefits of PA (suggested by Hall et al. [46], see [20] for a recent experimental study). The current study indicates that PA mediates the effects of age and executive functioning on memory performance because older adults who are more physically active could have higher executive functions, and consequently, better episodic memory. This suggests that the benefits of PA could be associated with a reorganization or preservation of cognitive processes.

In order to further explore the relation between PA, memory and executive functions, it would be interesting to investigate more precisely underlying cognitive processes to memory responses (e.g., using statistical analyses such as Multinomial Tree Processing) or indicators of accuracy in episodic memory performance. For example, it would be interesting to consider recognition accuracy using *Remember/Know* judgment or indicators of accuracy and strategies from the signal detection theory [47]. In fact, we assume that *Remember* responses would be higher in older adults who engage in the most PA, as these responses involve more conscious recollection and executive processes than *Know* responses. Moreover, according to the signal detection theory, older adults use a rather liberal strategy (more "old" responses), increasing the number of false alarms in this age group. We can therefore assume that for older adults who engage in the most PA, the liberal strategy would be less important and therefore memory performance would be more accurate because of fewer false alarms. Taking these strategies into account would provide new insights into the PA-memory relationship during aging.

This study focused on general executive functioning and was based on an index calculated from three measures capturing a fraction of each of the executive functions: one for inhibition, one for flexibility, and one for updating. It might be interesting to examine the effects of each of the executive functions, assessed each by different measures, in the relationship between age, memory and PA. It would also be relevant to consider that the nature of the PA might influence executive functions differently depending on the demands required by the PA. For example, physical activities involving "open" skills (i.e., in an unpredictable environment, e.g. table tennis, basketball) would provide greater benefits on cognition than those involving "closed" skills (i.e., in a predictable, stable environment, e.g. running, swimming) [48]. Moreover, executive functions play an important role in efficient mnemonic strategies [6] and memory performance [10, 11, 49]. Hence, if PA has a beneficial effect on executive performance, we can assume that this would in turn influence mnemonic strategies (e.g. [50]). Future studies should investigate changes in the use and efficacy of mnemonic strategies by physically active older adults during encoding and retrieval.

Finally, concerning PA, we believe that the use of questionnaires, validated with objective data (as Baecke questionnaire [37] validated by [38, 51]), enables PA to be assessed over longer periods (e.g., one year in the present study) than objective measures such as actimetry. It would thus be particularly interesting to develop the use of such questionnaires in large (e.g., epidemiological) studies. However, these questionnaires may also represent one of the limitations of this study. Indeed, the use of questionnaires can lead to over- or under-estimation of physical activity levels, masking possible differences between age groups. It is therefore essential to replicate these results, and all those concerning the benefits of physical activity on cognition, using other measures of physical activity levels, especially objective measures.

To conclude, while this study suggested that executive functioning is one of the many factors contributing to the benefits of PA on episodic memory, other explanations could be put forward and should be considered. These include the two-way link between PA and cognition, many studies demonstrating that people with higher executive performance are more apt to persevere in healthy behavior, such as PA [52, 53]. This would in turn improve executive functions. A two-directional model between episodic memory and PA has recently been proposed, highlighting the need for further investigation [54]. One of the main limitations concerns the cross-sectional design of our study. To conduct more robust mediation analyses that establish causal relationships, it is important to have interventional data [55]. The mediation model, which is suggested by cross-sectional data, could be confirmed and developed by interventional studies. If done, it would also be interesting to explore in greater depth the effects of different types of PA (e.g., aerobic, resistance), intensity and duration. While cerebral and cognitive factors are those that have been the most extensively studied, PA also leads to psychological changes (e.g., in stress, sleep quality, depression, and social interactions; [56–59]); further research is required to examine the effects of these changes on the benefits observed. Although there is a wealth of literature showing the benefits of PA on cognition, a better understanding of the underlying mechanisms is needed. Accordingly, as PA is one of several cognitive reserve factors, it would be interesting to replicate our results while controlling for others factor, as in Kachouri et al. [19], or examining a possible moderation effect of these other cognitive reserve factors on the effects of PA on memory. In this way, it would be possible to maximize the benefits of PA on cognitive performance by showing "how" these benefits exist and "who" benefits the most.

## Data Availability

This research was not preregistered. Data and analytic methods (R script) are freely available from Open Science Framework database (https://osf.io/kqnga/?view_only=0e9be42b76f24ad18744279623e05bc8).
